# Synthetic Biology: Emerging Concepts to Design and Advance Adeno‐Associated Viral Vectors for Gene Therapy

**DOI:** 10.1002/advs.202004018

**Published:** 2021-02-26

**Authors:** Hanna J. Wagner, Wilfried Weber, Martin Fussenegger

**Affiliations:** ^1^ Department of Biosystems Science and Engineering ETH Zurich Mattenstrasse 26 Basel 4058 Switzerland; ^2^ Faculty of Biology University of Freiburg Schänzlestraße 1 Freiburg 79104 Germany; ^3^ Signalling Research Centres BIOSS and CIBSS University of Freiburg Schänzlestraße 18 Freiburg 79104 Germany; ^4^ Faculty of Science University of Basel Klingelbergstrasse 50 Basel 4056 Switzerland

**Keywords:** AAV; adeno‐associated virus, capsid modifications, engineering, gene delivery, molecular switches, vector design

## Abstract

Three recent approvals and over 100 ongoing clinical trials make adeno‐associated virus (AAV)‐based vectors the leading gene delivery vehicles in gene therapy. Pharmaceutical companies are investing in this small and nonpathogenic gene shuttle to increase the therapeutic portfolios within the coming years. This prospect of marking a new era in gene therapy has fostered both investigations of the fundamental AAV biology as well as engineering studies to enhance delivery vehicles. Driven by the high clinical potential, a new generation of synthetic‐biologically engineered AAV vectors is on the rise. Concepts from synthetic biology enable the control and fine‐tuning of vector function at different stages of cellular transduction and gene expression. It is anticipated that the emerging field of synthetic‐biologically engineered AAV vectors can shape future gene therapeutic approaches and thus the design of tomorrow's gene delivery vectors. This review describes and discusses the recent trends in capsid and vector genome engineering, with particular emphasis on synthetic‐biological approaches.

## Graphical abstract

The concept of upgrading adeno‐associated viral (AAV) vectors by means of synthetic‐biological engineering is initiating a new era in gene therapy. The development of strategies to control and fine‐tune transduction mechanisms or transgene expression gives rise to a new generation of viral vectors. This review discusses recent trends in the field and particularly focuses on synthetic‐biological AAV engineering.

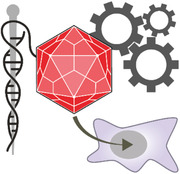

## 1 Introduction

Over 70 years after the idea of gene therapy was put down into writing for the first time,^[^ ^1^ ^]^ research endeavors in viral vehicle development have come to fruition: at least 13 viral‐based gene therapy products have been approved for the treatment of cancer or rare monogenic diseases to date.

Gene therapy aims at the transfer of therapeutic genes into a patient's cells to treat diseases on the genetic level.^[^ ^2^ ^]^ In most cases, the natural ability of viruses to enter cells and deliver genetic material is exploited to transfer therapeutic genes to target cells. Depending on the target cells, two different approaches are mainly applied: stem cells or dividing precursor cells are treated ex vivo with viral vectors, such as retroviral or lentiviral vectors, capable of stably inserting the therapeutic gene into the target cell genome. As a consequence, the therapeutic gene is preserved in progenitor cells. One major concern associated with integrating vectors is the potential for insertional mutagenesis.^[^ ^3^ ^]^ Postmitotic, nondividing cells are therefore typically targeted in vivo by nonintegrating vectors derived from, e.g., adeno‐associated virus (AAV), adenovirus, or herpes simplex virus.^[^ ^4^, ^5^, ^6^ ^]^ The genetic information encoded in these vectors is maintained as episomal DNA for prolonged periods of time.

With three approvals and over 100 ongoing clinical trials, AAV vectors have become one of the leading gene delivery vehicles for gene therapy. In 2012, Alipogene tiparvovec (Glybera) was the first AAV therapy approved in Europe for the treatment of lipoprotein lipase deficiency.^[^ ^7^ ^]^ Although the drug was not commercially successful due to low demand and its extremely high cost and is therefore no longer on the market, it clearly demonstrated the safety and functionality of AAV‐based therapeutic approaches. Since then, Voretigene neparvovec (Luxturna) for the treatment of Leber's congenital amaurosis and Onasemnogene abeparvovec (Zolgensma) for the treatment of spinal muscular atrophy received U.S. Food and Drug Administration (FDA) approval in 2017 and 2019, respectively.^[^ ^8^, ^9^ ^]^ A major factor in the success of AAV as a therapeutic gene delivery vehicle is its low immunogenicity and lack of pathogenicity.^[^ ^10^ ^]^ Current clinical research focuses on the treatment of monogenetic diseases by gene replacement, gene silencing, or gene editing, as well as on gene addition approaches using AAV‐based vectors (for a comprehensive overview, see the recent review by Wang et al.^[^ ^11^ ^]^). AAV also holds promise for cancer therapies,^[^ ^12^, ^13^, ^14^, ^15^, ^16^, ^17^ ^]^ the development of novel types of vaccines,^[^ ^18^, ^19^, ^20^, ^21^ ^]^ and vectored immunoprophylaxis.^[^ ^22^, ^23^, ^24^, ^25^, ^26^ ^]^

In recent years, increasing knowledge of the structure and biology of AAV has led to the targeted modification of its proteinaceous capsid, which has opened the door to viral vectors with enhanced tissue specificity, cellular uptake, transgene expression, and protection from neutralizing antibodies. The engineering of the capsid has been complemented by sophisticated design of the packaged vector genome to modulate the time, location, and strength of transgene expression. In addition, synthetic‐biological principles have extended the toolbox for upgrading AAV‐based vectors at various levels of the transduction cycle.

In this review, we summarize recent advances in the engineering of recombinant AAVs (rAAVs) with an emphasis on synthetic‐biological approaches. We will first give an overview of the biology and infection cycle of AAV. Then, we will describe engineering strategies to control distinct stages of AAV transduction—at the capsid and at the vector genome level. Although the therapeutic potential of most of these designed vectors has yet to be evaluated in the clinical setting, they represent novel concepts that will likely contribute to both the development of advanced gene delivery vehicles and the uncovering of fundamental AAV biology‐related mechanisms.

## 2 AAV—A Small but Promising Gene Delivery Vehicle

AAVs are small (≈25 nm in diameter), nonenveloped, single‐stranded DNA (ssDNA) viruses. They are classified as dependoviruses—a genus belonging to the family of *Parvoviridae* (“parvum”: Latin for small).^[^ ^27^ ^]^ As the genus name implies, AAV replication and production of progenitor viral particles depend on coinfection with a helper virus such as adenovirus, herpes virus, or vaccinia virus.^[^ ^28^, ^29^, ^30^ ^]^ In the absence of these helper viruses, latent infection with AAV occurs by integration into the host genome, preferentially at the AAVS1 locus on chromosome position 19q13.^[^ ^31^, ^32^ ^]^

### 2.1 Genome Organization

**Figure 1:**
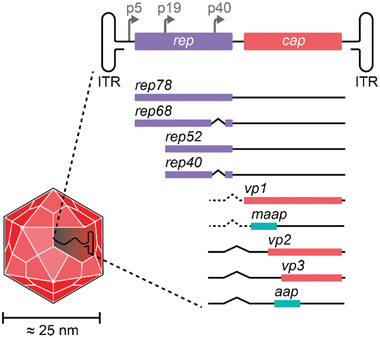
Genomic organization of AAV. The AAV capsid contains an ≈4.7 kb ssDNA genome comprised of inverted terminal repeats (ITRs) flanking the regulatory regions and coding sequences for AAV replication. The two promoters p5 and p19 drive the expression of two *rep* transcripts, which are alternatively spliced and result in the production of two large (Rep68 and Rep78) and two small (Rep40 and Rep52) proteins. The p40 promoter controls the expression of the *cap* transcript. Alternative splicing, nonconventional start codons, and the use of alternative reading frames result in five gene products: the three capsid proteins (VP1, VP2, and VP3), the assembly‐activating protein (AAP), and the membrane‐associated accessory protein (MAAP).

The 4.7 kb ssDNA genome of AAV is encapsidated in both (+) and (−) single‐strand orientation. It is composed of inverted terminal repeats (ITRs), which are structural elements required for second strand synthesis, genome amplification, integration into the chromosome, and ssDNA packaging. The ITRs flank the four genes *rep*, *cap*, *aap*, and the newly identified *maap* ^[^ ^33^ ^]^ (**Figure** **1**). The *rep* gene encodes four nonstructural proteins of different molecular weight (Rep40, Rep52, Rep68, Rep78). They are produced from two alternatively spliced mRNAs whose expression is controlled by the two promoters p5 and p19 (Figure 1). The promoter numbers indicate the location on the AAV genome in map‐units (1 map unit corresponds to 1% of the genome size in bp). The Rep proteins are important regulators of AAV integration, reactivation, replication, and genome packaging. Expression of the *cap* gene, which is controlled by the p40 promoter, results in the production of three structural proteins, VP1 (viral protein‐1; 87 kDa), VP2 (72 kDa), and VP3 (62 kDa) (Figure 1). VP3 and VP2 share identical sequences with the carboxy‐terminus of VP1. These proteins are produced in a 1:1:10 ratio (VP1:VP2:VP3) from two alternatively spliced mRNAs and assemble an icosahedral capsid composed of 60 VPs. VP1 is produced from the minor splice product. The translation of VP3 is initiated from a conventional AUG start codon, whereas low levels of VP2 are produced from a nonconventional ACG start site. In addition to the VP proteins, the *cap* region encodes the assembly‐activating protein (AAP) in an alternative open reading frame with a nonconventional CUG start codon.^[^ ^34^ ^]^ AAP localizes in the nucleus, where it plays an essential role for most serotypes (with the exception of AAV4, AAV5, and AAV11^[^ ^35^ ^]^) in promoting the capsid assembly of newly synthesized viral proteins.^[^ ^34^ ^]^ Recently, an intriguing study employed a codon‐scanning approach to detect hidden genes in alternative reading frames.^[^ ^33^ ^]^ The authors identified an additional gene product in the VP1 region coding for a membrane‐associated accessory protein (MAAP) with an as‐yet undefined function. Interestingly, individual MAAP mutations had no negative impact on viral production, but production of MAAP mutants was markedly impaired in the presence of a competitive wild‐type AAV genome. In addition, the authors questioned the existence of the gene X^[^ ^36^ ^]^ putatively encoded on an alternative reading frame in the 3’ region of *cap*.

### 2.2 Tropism

Up to date, more than ten AAV serotypes have been isolated from human and nonhuman primate tissues. In general, these serotypes show a broad tropism, though the different serotypes have preferences for distinct tissues. The cell and tissue specificities of different serotypes can be harnessed in the development of therapeutic vectors. For example, AAV8 efficiently transduces hepatocytes in mice and nonhuman primates upon systemic administration and is thus a preferred vehicle for gene delivery to the liver.^[^ ^37^, ^38^ ^]^ AAV1 shows a skeletal and cardiac muscle tropism and was used for Glybera to deliver a gene encoding a gain‐of‐function variant of the lipoprotein lipase (LPL^S447X^) to muscle cells for the treatment of LPL deficiency.^[^ ^39^, ^40^ ^]^ AAV9 preferentially binds to galactose as its primary receptor, which is thought to contribute to its ability to cross the blood–brain barrier.^[^ ^41^, ^42^ ^]^ As a result, this serotype efficiently targets motor neurons upon intravenous administration, and it has been successfully used in clinical trials for gene delivery to the brain and spinal cord in the case of the recently approved Zolgensma.^[^ ^9^, ^43^ ^]^ Moreover, AAV9 efficiently infects muscle tissues. Consequently, this serotype is a preferential choice for muscle‐directed gene therapies in preclinical and clinical trials.^[^ ^11^ ^]^ So far, most AAV serotypes have been discovered in contaminated adenovirus stocks, human clinical samples, or primates.^[^ ^44^ ^]^ However, the identification and investigation of AAV isolates from other animal species has also attracted interest with respect to their tropism, serological profile, and compatibility with human tissues. For example, a recent study reported a porcine AAV isolate, AAVpo1, that robustly transduces muscle tissues and, in comparison with AAV9, is remarkably detargeted from the liver.^[^ ^45^, ^46^ ^]^

In addition to the natural serotypes, a number of designer capsids with enhanced tissue tropism have been generated by means of rational and evolutionary approaches (see Sections 3 and 4).

### 2.3 Infection Cycle

#### 2.3.1 Receptor Binding

**Figure 2:**
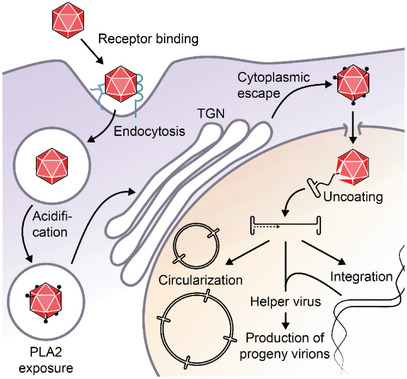
Model of AAV infection. Upon binding to its cellular receptors, AAV is internalized by endocytosis. Acidification of the endosome results in conformational changes of the AAV capsid and the exposure of a phospholipase A2 (PLA2) domain. After trafficking to the Golgi apparatus and escape to the cytoplasm, AAV enters the nucleus via the nuclear pore complex. In the nucleus, the AAV genome is uncoated and converted to dsDNA. In the absence of helper functions, the dsDNA is integrated into the genome to establish a latent infection. The presence of a helper virus stimulates the replication and production of progenitor AAV virions. In contrast to wild‐type AAV, rAAV lacking the genetic information for the Rep proteins maintains long‐term transgene expression through circularization and high‐molecular‐weight circular concatemer formation.

The differences in tropism are caused by variations in the capsid surface, which affects the preference for distinct cellular receptors. The initial cellular attachment is mediated by primary receptors, which are mainly glycan structures that are expressed by many cell types (**Figure** **2**). Subsequent endocytosis is facilitated by secondary receptors such as integrins (*α*v*β*5 integrins for AAV2)^[^ ^47^, ^48^ ^]^ or growth factor receptors (fibroblast growth factor receptor (FGFR) and hepatocyte growth factor receptor (HGFR) for AAV2;^[^ ^49^, ^50^ ^]^ epidermal growth factor receptor for AAV6;^[^ ^51^ ^]^ or platelet‐derived growth factor receptor (PDGFR) for AAV5^[^ ^52^ ^]^). Although these receptors have been described as co‐receptors for AAV infection, recent knockout experiments suggest they may have a rather modest role. In contrast, KIAA0319L (termed AAV receptor, AAVR) is widely expressed in many tissues^[^ ^53^ ^]^ and has been identified as an essential entry factor for multiple AAV serotypes.^[^ ^54^ ^]^

#### 2.3.2 Endosomal Trafficking

Following internalization, AAV is transported by microtubules within endosomal compartments to the perinuclear region (Figure 2).^[^ ^55^ ^]^ Acidification of late endosomes results in conformational changes in VP1 and VP2, leading to exposure of the unique N‐terminus of VP1 (VP1u), which contains a phospholipase A2 domain that supports the escape of AAV virions from the endosomal system, and a nuclear localization signal for transport into the nucleus.^[^ ^56^, ^57^, ^58^ ^]^ The low pH environment in late endosomes has been associated with autocleavage of the AAV capsid.^[^ ^59^ ^]^ Moreover, endosomal proteases play a pivotal role in priming the subsequent nuclear uncoating, and thus infectivity, of many viruses. Cathepsin B and cathepsin L are important for efficient transduction by AAV2 and AAV8.^[^ ^60^ ^]^ These proteases bind to and cleave the capsids of both serotypes, which leaves the virions intact, but presumably promotes the conformational change of the capsid and the exposure of VP1u, priming AAV for the downstream infection steps.^[^ ^60^ ^]^ In addition, VP1u exerts proteolytic activity against disordered proteins at neutral pH.^[^ ^61^ ^]^ However, the role of this function during the viral life cycle remains to be clarified.

Recently, a genome‐wide CRISPR screen using the AAVR‐independent serotype rh32.33 identified the G‐protein coupled receptor GPR108 as another essential entry factor.^[^ ^62^ ^]^ Since GPR108 engagement depends on VP1u exposed in acidified endosomes, it seems likely that GPR108 supports AAV infectivity at the level of endosomal escape or trafficking, rather than cell attachment. Indeed, GPR108 localizes primarily to the Golgi compartment and is expressed almost ubiquitously by most cell types.^[^ ^63^ ^]^ Interestingly, GPR108 is not essential for AAV5, suggesting the existence of serotype‐specific differences in the transduction processes.^[^ ^62^, ^63^ ^]^

#### 2.3.3 Translocation to the Nucleus and Replication

After retrograde transport to the Golgi apparatus and escape into the cytoplasm, intact virions travel through the nuclear pore complexes into the nucleus, where uncoating of the AAV genome takes place (Figure 2).^[^ ^64^, ^65^ ^]^ Subsequently, the ssDNA genome is converted into dsDNA. At this point and in the absence of a helper virus coinfection, AAV establishes a latent infection by integrating its dsDNA into the host chromosome and silencing its genome by Rep‐mediated autorepression of the p5 promoter.^[^ ^31^, ^32^, ^66^, ^67^, ^68^ ^]^ Alternatively, the presence of a helper virus directly stimulates transcription and replication of the AAV genome and generates a replication‐promoting environment within the host cell. This includes the induction and freezing of the cell's productive synthesis (S) phase, inhibition of apoptosis, and the shut‐off of host gene expression.^[^ ^69^ ^]^ The ITRs of AAV provide 3’OH primers for polymerase *δ*‐mediated rolling hairpin replication of the AAV genome.^[^ ^70^, ^71^, ^72^ ^]^ Additionally, they contain binding sites for the large Rep proteins, whose helicase activity and endonuclease activity support the replication process.^[^ ^73^, ^74^ ^]^ The small Rep proteins channel the newly synthesized single‐stranded AAV genomes into preassembled capsids through the pore of the fivefold symmetry axis.^[^ ^75^, ^76^ ^]^ In the case of replication‐deficient, recombinant AAV vectors (rAAVs), the vector genomes circularize and form high‐molecular‐weight concatemers through intermolecular recombination (Figure 2).^[^ ^77^, ^78^ ^]^ These episomal DNA structures pose a low risk of genotoxicity and mediate long‐term transgene expression in nondividing cells, which are both important features for safe and efficient gene therapy.

### 2.4 AAV Vectors

**Figure 3:**
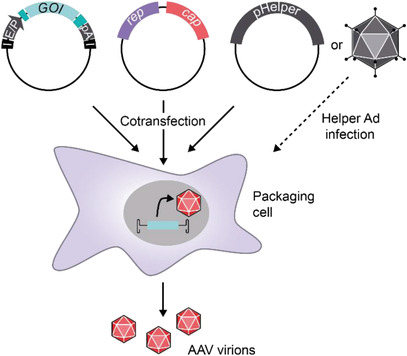
rAAV production in mammalian producer cell lines. Typically, human embryonic kidney cells are transfected with three plasmids. The vector plasmid (left) contains the expression cassette for the gene of interest (GOI) flanked by the AAV ITRs (I). Regulatory sections such as enhancers and promoters (E/P), polyadenylation sites (pA), and additional posttranscriptional regulatory elements (dark turquoise) fine‐tune the transgene expression. The rep‐cap plasmid (middle) produces the viral proteins, but is not packaged due to the lack of ITRs. The adenoviral helper function is provided by co‐transfection of a pHelper plasmid (right) or by infection with, e.g., adenovirus.

As mentioned above, the ITRs represent structural *cis*‐acting DNA elements required for replication and packaging of the AAV genome. Typical AAV‐based vector systems are generated by flanking transcriptional units of interest with ITRs and providing the genetic information of *rep* and *cap* in *trans* (**Figure** **3**). The helper virus functions required for high‐titer production of AAV can be provided either by infecting the producer cell line with a helper virus such as adenovirus or by co‐transfecting a plasmid encoding adenoviral helper genes (Figure 3).

Cloning the AAV genes and vector genome into plasmids facilitates their genetic manipulation. Over the past decade, sophisticated expression cassettes have been designed that contain fine‐tuned regulatory elements to restrict the expression of transgenes to distinct tissues, to adjust the level of transcription, or to enable control of expression by external stimuli (see Section 5.2). Moreover, the possibility of modifying the *cap* gene opened the door to engineer AAV capsids with altered or new functions in the AAV transduction process. Target functions include cellular uptake, nuclear transport, and transgene expression, and will be described in the following sections.

Upon systemic administration, AAV vectors transduce tissues and cells according to their capsid serotype. Over the last few years, research has aimed at enhancing the vector's ability to target distinct cells and tissues. This has often been combined with attempts to decrease the potential for inactivation by neutralizing antibodies (NAbs), which pose a major barrier to AAV's transduction capability in vivo. The problem of NAbs is illustrated by the fact that, depending on the AAV serotype, the geographic region, and the subjects’ age, up to 70% of the human population has encountered AAV before and thus has established a protective humoral immune response.^[^ ^79^, ^80^ ^]^ In the case of gene therapy, treatment of individuals exhibiting NAbs is expected to be inefficient, and consequently, such patients are excluded a priori from clinical trials.

Improvement of transduction specificity and the ability to escape from NAbs has been tackled by rational capsid engineering and evolutionary approaches.

## 3 Directed Evolution of AAV Capsid Variants

**Figure 4:**
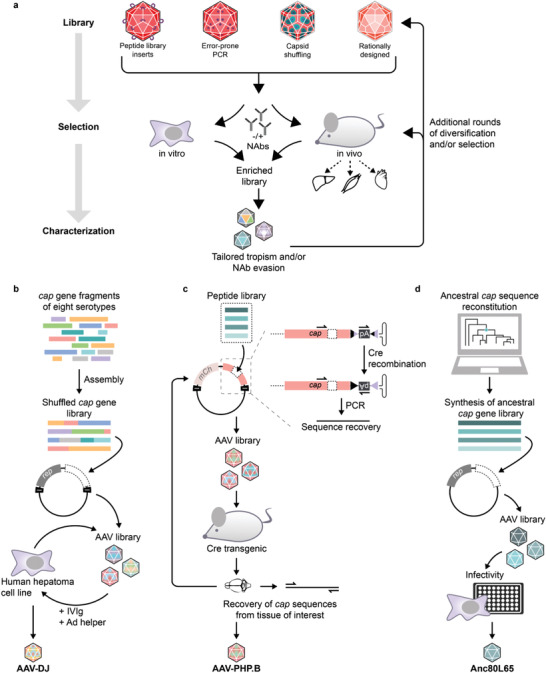
Directed evolution of tailored AAV variants. a) Capsid libraries are generated by the insertion of randomized peptides into exposed loop structures, by error‐prone polymerase chain reaction (PCR), by capsid shuffling, or by rational design. These libraries are subjected to a selection process based on their capability to transduce target cells in vitro or target tissues in vivo. Optionally, neutralizing antibodies (NAbs) are included to select capsids with both tailored tropism and the ability to evade NAbs. b) Generation of AAV‐DJ. The *cap* genes of eight AAV serotypes were fragmented, reassembled to a library of shuffled *cap* genes, and inserted into an ITR‐carrying *rep*‐*cap* plasmid for viral library production. Five rounds of amplification on a human hepatoma cell line in the presence of IVIg resulted in enrichment of a single variant, AAV‐DJ, with superior transduction efficiency.^[^ ^83^ ^]^ c) Cre recombination‐based AAV targeted evolution (CREATE) allows the selective recovery of library sequences from Cre‐expressing target cells. For the generation of a CNS‐targeted AAV variant, a peptide library was cloned into an AAV9 vector plasmid harboring a floxed polyadenylation sequence (pA). Cre‐mediated inversion of pA results in a template for sequence recovery by PCR. Two rounds of selection in transgenic mice expressing Cre in astrocytes led to the isolation of CNS‐targeted AAV‐PHP.B from brain and spinal cord tissue.^[^ ^88^ ^]^ d) Identification of the ancestral capsid variant Anc80L65 for gene delivery. The in silico reconstruction of the evolutionary AAV lineage enabled the design and synthesis of a *cap* library covering the probable sequence space of an ancestral AAV variant. Screening of assembly and transduction efficiency led to the identification of Anc80L65, which is characterized by broad tissue tropism and resistance to antisera raised against other serotypes.^[^ ^89^ ^]^

Evolutionary approaches use capsid libraries that have been generated by random mutagenesis,^[^ ^81^, ^82^ ^]^ capsid serotype shuffling,^[^ ^83^, ^84^ ^]^ or peptide library insertion^[^ ^85^, ^86^, ^87^ ^]^ (**Figure** **4**). AAV variants with the desired tropism are enriched by transducing target cells in vitro or in vivo. The addition of NAbs, typically in the form of pooled human intravenous immunoglobulins (IVIg), during the selection process supports the selection of NAb‐evading AAVs (Figure 4).

### 3.1 Directed Evolution by Random Mutagenesis

Random mutagenesis of the *cap* gene by error‐prone PCR has been used to generate AAV variants with enhanced transduction^[^ ^90^ ^]^ and NAb evasion^[^ ^81^, ^91^ ^]^ properties. These studies have also contributed to the identification of positions on the capsid surface that tolerate amino acid substitutions. The increasing understanding of the capsid fitness landscape has significantly facilitated the rational engineering of the AAV surface (see Section 4).

### 3.2 Capsid Shuffling

Another evolutionary approach generates chimeric capsids by shuffling *cap* segments of different serotypes.^[^ ^83^, ^84^, ^92^ ^]^ A noteworthy example is the development of AAV‐DJ—an AAV2/8/9 chimera that has been generated by shuffling the capsids of eight serotypes and performing in vitro selection with liver cells in combination with negative selection using IVIg (Figure 4b).^[^ ^83^ ^]^ Compared to other wild‐type AAV serotypes, AAV‐DJ shows superior transduction of both hepatic and nonhepatic cell lines in vitro and performs efficiently in vivo. The versatility of this engineered serotype has been demonstrated by many examples as, for instance, the gene delivery to fetal pig fibroblasts,^[^ ^93^ ^]^ genome editing in human keratinocytes,^[^ ^94^ ^]^ and transduction of liver ductal organoids.^[^ ^95^ ^]^ Moreover, in vivo applications of AAV‐DJ in animal models include the transduction of murine taste cells,^[^ ^96^ ^]^ HIV‐1 provirus excision in HIV‐1 Tg26 transgenic mice,^[^ ^97^ ^]^ the generation of an animal model to study the pathogenesis of Huntington's disease,^[^ ^98^ ^]^ and gene therapy approaches for the treatment of femoral fractures^[^ ^99^ ^]^ or acute myocardial ischemia.^[^ ^100^ ^]^ These examples highlight the broad tropism of AAV‐DJ, which goes beyond the initially intended liver‐specific transduction, but might be advantageous for applications requiring the transduction of many cell types and tissues. Interestingly, recent reports question the use of this serotype for the transduction of primary human hepatocyte populations in mice.^[^ ^101^, ^102^ ^]^ In these studies, the engineered serotypes NP40 and NP59, that had been selected in a mouse xenograft model of the human liver, outperformed AAV‐DJ. The reasons for the discrepancy in mouse and human liver transduction still need to be clarified but likely involve processes after uncoating such as second‐strand synthesis and transcription.^[^ ^101^, ^103^ ^]^ In contrast, ex vivo treatment of primary human liver organoids revealed comparable transduction efficiencies for AAV‐DJ, NP40, and NP59, suggesting primary cell line‐specific differences and/or an impact by the animal model. Of note, the above‐mentioned xenograft experiments included only female mice for the assessment of the performance of AAV‐DJ.^[^ ^101^, ^102^ ^]^ Since the transduction pattern in the liver of mice is sexually dimorphic, it would be interesting to compare the performance of AAV‐DJ in mouse xenograft models of both sexes.^[^ ^104^ ^]^

Coupling error‐prone mutagenesis with capsid shuffling can increase the diversity of capsid libraries. This strategy has been used for the generation of AAV vectors capable of transducing human pulmonary epithelium, which offers the potential of treating monogenetic respiratory diseases.^[^ ^105^ ^]^

Much effort has also been directed to the development of viral vectors targeting cell types of the nervous system. AAV is a promising candidate because it efficiently transduces neural cells. As mentioned above, AAV9 uses galactose as a primary receptor that is presumably associated with its ability to cross the blood–brain barrier.^[^ ^41^, ^106^ ^]^ Although this property may be exploited to circumvent the need for direct administration into the central nervous system, the broad tropism of AAV poses a major obstacle due to associated risks of off‐target transduction, side effects, and loss of efficiency. Recently, oligodendrocyte‐targeted AAVs have been generated by capsid shuffling and in vivo selection and resulted in a vector variant (Olig001) with high cell‐specificity and low peripheral tissue tropism.^[^ ^107^ ^]^

### 3.3 Peptide Libraries

Neither PCR‐based mutagenesis nor capsid shuffling requires knowledge of the structure and infection cycle of AAV. However, since modifications span the whole *cap* gene the likelihood of mutations that affect capsid assembly and/or infectivity must be considered. An alternative evolutionary approach for the generation of modified AAV vectors is based on peptide libraries inserted into surface‐exposed capsid loops. Devermann et al. developed an elegant approach for brain‐targeted AAV vectors, using a peptide library of AAV9 harboring an rAAV genome with a floxed polyadenylation sequence (pA) (Figure 4c).^[^ ^88^ ^]^ Upon transduction of Cre‐positive cells, pA is inverted and generates a template for PCR amplification with appropriately designed primers. This so‐called Cre recombination‐based AAV‐targeted evolution (CREATE) was used to enrich AAV vectors that can cross the blood–brain barrier and efficiently transduce the central nervous system (CNS) upon intravenous administration in mice expressing Cre in astrocytes (Figure 4c).^[^ ^88^ ^]^ One construct, AAV‐PHP.B, showed superior CNS targeting and reduced transduction of peripheral tissues compared to the parental AAV9. Furthermore, CREATE was adapted for multiplexed viral capsid selection (M‐CREATE).^[^ ^108^ ^]^ The use of next‐generation sequencing enabled the correction for over‐represented sequences accumulated during DNA‐ and AAV‐library production, the sequence analysis of viral DNA extracts from different tissues, and thus the determination of enrichment factors and enriched variants in selected tissues. The in‐depth sequence analysis of this approach allowed cluster analysis and the deduction of amino acid sequence logos for variants enriched in distinct target tissues.^[^ ^108^ ^]^ This strategy identified AAV variants that efficiently crossed the blood–brain barrier and exhibited an enhanced tropism toward cells of the central nervous system.^[^ ^108^ ^]^ Other peptide libraries have been successfully used for evolving AAV vectors specific for brain microvasculature endothelial cells (AAV‐BR1^[^ ^86^ ^]^), human keratinocytes,^[^ ^109^ ^]^ or retrograde axonal transport in distinct neurons.^[^ ^110^ ^]^

### 3.4 Pooled AAV Libraries

The combination of different types of AAV capsid libraries increases the overall size and diversity of the library. Dalkara et al. pooled libraries consisting of randomized peptide insertions, shuffled capsid variants, error‐prone mutants, and randomized loop sequences.^[^ ^111^ ^]^ Several rounds of transduction, *cap* recovery, and re‐packaging enriched AAVs capable of reaching the outer retina from the vitreous.^[^ ^111^ ^]^ Interestingly, isolated AAVs were almost exclusively derived from the peptide insertion library.^[^ ^111^ ^]^ The resulting AAV2.7m8 efficiently transduced the outer retina of mice and the fovea of nonhuman primates. In another study, Isgrig et al. used AAV2.7m8 as a gene delivery vehicle to cochlear inner and outer hair cells.^[^ ^112^ ^]^ The potential of this synthetically evolved capsid variant is currently being assessed in phase I and phase II clinical trials for the treatment of age‐related macular degeneration and diabetic retinopathy, respectively (**Table** **1**). Tervo et al. applied a similar pooled library approach for the enrichment of AAV variants that retrogradely access projection neurons.^[^ ^113^ ^]^ Here, only combinations with peptide insertions and point mutations resulted in enriched AAV variants.

**Table 1:** Clinical trials with capsid‐engineered AAV

AAV serotype	Capsid modification	Transgene	Company	Condition	Phase	ClinicalTrials.gov identifier
AAV2	Three Tyr to Phe mutations	hCNGB3	Applied Genetic Technologies Corp.	Achromatopsia	Phase II	NCT 02599922
AAV2	^588^LALGETTRP insertion; V708I	Aflibercept	Adverum Biotechnologies	Oedema, macular, Diabetic	Phase II	NCT 04418427
				Macular degeneration, age‐related, wet	Phase I	NCT 03748784
AAVS3	Not disclosed^[^ ^101^ ^]^	hFIX variant	Freeline Therapeutics	Hemophilia B	Phase III	NCT 03641703
AAV‐LK03^[^ ^102^ ^]^ (Spark200)	Chimeric capsid closely related to AAV3B; generated by capsid shuffling	hFVIII variant	Spark Therapeutics	Hemophilia A	Phase III/IV	NCT 03876301 (IV)
Spark100	Not disclosed^[^ ^103^ ^]^	FIX‐Padua	Spark Therapeutics	Hemophilia B	Phase III	NCT 03861273

### 3.5 Backward‐Directed Evolution of AAV Variants

The above‐described examples highlight the power of forward‐directed molecular evolution for the development of tailored AAV variants. A complementary and highly intriguing approach is the backward‐directed molecular evolution of AAV capsids. Zinn et al. used in silico phylogenetic and statistical modeling to infer ancestral capsid sequences (Figure 4d).^[^ ^89^ ^]^ The reconstitution and testing of a library covering the sequence space of Anc80—a common ancestor of many naturally occurring as well as clinically relevant AAVs—resulted in the identification of Anc80L65 (Figure 4d).^[^ ^89^ ^]^ This AAV variant is 8.6% divergent from AAV.rh10, which is the closest contemporary serotype. Anc80L65 exhibits high thermostability, satisfying production titers, and efficient gene delivery to the retina, liver, and muscle in mice as well as to the liver in nonhuman primates.^[^ ^89^, ^114^ ^]^ Surprisingly, Anc80L65 efficiently transduces difficult‐to‐target cells in mice, including inner and outer hair cells in the adult cochlea and kidney mesenchymal cells.^[^ ^115^, ^116^ ^]^ Moreover, the lack of AAV‐related toxicity and the low cross‐reactivity of rabbit and murine antisera raised against other contemporary serotypes demonstrate the high potential of this ancestral serotype for clinical applications.^[^ ^89^ ^]^ However, similar to other designer AAV variants, the data collected in vitro and in animal models still need to be validated in human models.

### 3.6 Challenges in Directed Evolution of AAVs

Regardless of the type of library used, one important requirement in screening is the coupling of genotype and phenotype. In the case of AAV, this is mainly achieved by transfecting the library plasmid at low concentrations. Previous studies suggest a so‐far poorly understood correlation between the identity of the capsid and the packaged genome.^[^ ^117^ ^]^ Schmit et al. conducted a comprehensive study evaluating the effect of plasmid library dilution on the cross‐packaging and capsid mosaicism of virions.^[^ ^118^ ^]^ A decrease in plasmid amount per cell led to a marked decrease in cross‐packaging, which correlated with the degree of capsid mosaic formation. Interestingly, AAV titers remained relatively constant across dilutions. This study emphasizes the importance of titrating AAV plasmid libraries to achieve efficient virion production, low cross‐packaging, and low capsid mosaicism.^[^ ^118^ ^]^ Another important factor in AAV research is the differential performance of virus variants in distinct species. Research generated with mouse models does not necessarily translate to humans or other animal models such as nonhuman primates. This point is also critical for in vivo evolution of AAV variants, and was strikingly demonstrated in the case of AAV PHP.B, which shows excellent transduction of the CNS in C57BL/6J mice, but shows relatively poor CNS tropism in mouse strains lacking the GPI‐anchored surface protein LY6A (e.g., Balbc/J) and in nonhuman primates.^[^ ^119^, ^120^ ^]^ Overcoming this limitation of evolutionary approaches performed in vivo will require more sophisticated enrichment strategies, such as combinations of in vitro and in vivo selection using different cell lines and species, and underlines the importance of preclinical studies with animal model surrogates for human patients.

### 3.7 New Techniques and Methodologies for Directed Evolution of AAVs

In recent years, the construction of evolved AAV variants has been facilitated by the development of new techniques and high‐throughput methodologies. Especially, DNA barcoding of AAV coupled with next‐generation sequencing has facilitated the characterization of individual AAV variants with respect to, e.g., virion formation, transduction efficiency, blood clearance, and neutralizing epitopes.^[^ ^121^ ^]^ Davidsson et al. extended this approach to enable barcoded rational AAV vector evolution screening, termed BRAVE.^[^ ^110^ ^]^ They designed an AAV production plasmid containing an ITR‐flanked GFP expression cassette with a barcode in the 3’ UTR and *cis*‐acting Rep‐Cap. The insertion of loxP sites enabled Cre recombination‐mediated linkage of the capsid variant to the barcode, which could be evaluated by next‐generation sequencing. The decoupling of the *cap* gene from the vector genome and the sequencing of expressed barcode mRNA add layers of flexibility, including the possibility to screen for AAV variants that undergo the full transduction cycle.

Recently, Ogden et al. presented an intriguing study that characterized all possible mutations in the AAV2 *cap* gene.^[^ ^33^ ^]^ More specifically, they tested the effects of all possible amino acid substitutions, insertions, deletions, and stop codons on viral production and in vivo biodistribution. The resulting capsid fitness landscape illustrates tolerated and deleterious mutations across the *cap* gene, and will be extremely helpful for future rational capsid engineering and library design. Moreover, the effects on neutralization with the A20 antibody and on thermal stability were investigated. In addition, stop codon screening in alternative reading frames among synonymous capsid sequences revealed three notable findings: first, nonsense mutations in the open reading frame (ORF) of the *aap* gene impair virion production, which underlines the role of the gene product in capsid assembly. Second, screening of the previously proposed gene X revealed no effect on the fitness of AAV. Third, a new gene was identified in a +1‐shifted ORF in VP1. The product of this gene, the so‐called membrane‐associated accessory protein (MAAP), seems to be involved in the so‐far‐inexplicable genome–phenotype coupling of AAV capsid libraries.^[^ ^33^ ^]^ The screening of additional serotypes might uncover mutual relationships as well as unique characteristics influencing viral fitness, and could help in generating optimized AAV variants.

## 4 Redirection of AAV by Rational Engineering of the Capsid

**Figure 5:**
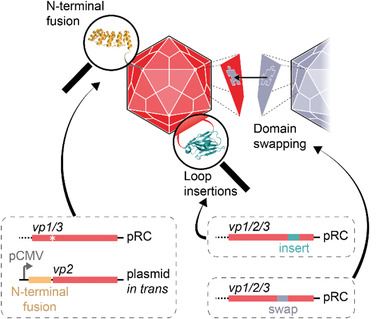
Rational engineering of the AAV capsid. Peptides or small protein domains can be inserted into surface‐exposed loops. Larger protein domains are preferably fused to the N‐terminus of VP2. The resulting fusion construct is provided in trans to the *rep*‐*cap* plasmid (pRC). Optionally, the start codon of VP2 is mutated in pRC (asterisk), which prevents the incorporation of unmodified VP2 into the capsids. Desirable capsid features from other AAV serotypes can be transferred by domain swapping.

Compared to evolutionary approaches resulting in altered or enhanced tissue specificity of AAV, rational approaches based on fundamental research combined with insights from evolutionary engineering have opened the door to truly redirected viral vectors that are specific for distinct cellular receptors. Three main strategies are applied for equipping capsids with new functions (**Figure** **5**). The first strategy uses surface‐exposed loops for the integration of peptide ligands or small protein domains. The second fuses protein domains to the N‐terminus of the capsid protein VP2, and provides this construct in *trans* to the *rep*‐*cap* plasmid. The third strategy transfers capsid properties from other serotypes by domain swapping. The following sections describe these strategies in more detail and give an overview of the latest developments.

### 4.1 Modification of Surface‐Exposed Loops

The idea of modifying the capsid of AAV2 to allow rational targeting of distinct cell types emerged over 20 years ago, before the crystal structure of AAV had been solved. Girod et al. identified position 587 in the variable region VIII (the so‐called G12/G13‐loop^[^ ^122^ ^]^) of the AAV2 capsid as a suitable site for the insertion of an integrin binding peptide, which efficiently retargeted AAV2 to integrin‐expressing cells.^[^ ^123^ ^]^ Since then, modification of this site has enabled, e.g., the targeting of distinct cell types,^[^ ^124^, ^125^ ^]^ the affinity purification of viral vectors,^[^ ^17^ ^]^ the display of enzymes,^[^ ^126^ ^]^ and the insertion of peptide libraries for directed evolution (see Section 3). Importantly, this most frequently used site of insertion in AAV2 lies between R585 and R588, which are both involved in primary receptor (heparan sulfate) binding.^[^ ^127^, ^128^ ^]^ Previous work suggests that the flexibility, size, and charge of inserts all influence the positioning and accessibility of R585 and R588, and their ability to mediate heparan sulfate binding.^[^ ^126^, ^129^ ^]^

Position 453 lies in the variable region IV (VR IV) located in the highest protrusion of AAV2 (the GH2/GH3 loop) and has been identified as an alternative to the 587 insertion site.^[^ ^130^ ^]^ Notably, the insertion of an integrin‐binding peptide at position 453 was influenced by residues R585 and R588 and the product was only functional in combination with arginine‐to‐alanine mutations.^[^ ^130^ ^]^ An alternative approach inserted leucine zippers at VP position 453 of AAV9 to noncovalently attach peptides to the capsid.^[^ ^131^ ^]^ Although the high molar excess of complementary peptide required for capsid functionalization calls for optimization, the system might offer a universal and modular tool for modifying the AAV surface.

**Figure 6:**
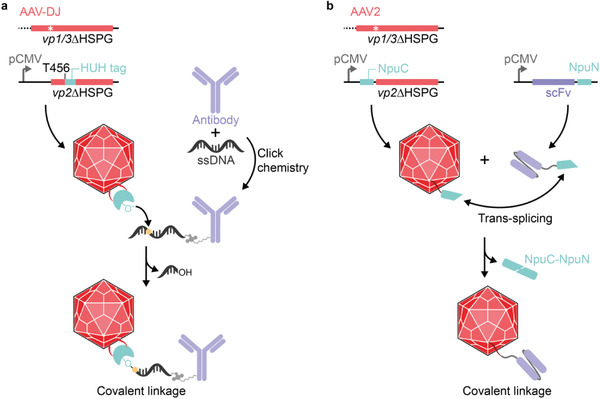
Covalent coupling of ligands to surface‐displayed protein domains. a) Covalent coupling of ssDNA‐tagged antibodies to HUH tag‐displaying AAV‐DJ. The HUH tag was inserted into the variable region VI (position T456 of AAV‐DJ) of receptor‐blinded VP2 (*vp2ΔHSPG*) and expressed in trans to the receptor‐blinded *rep*‐*vp1/3* plasmid harboring a mutated start codon for *vp2* (white star) for the production of HUH tag‐displaying AAV‐DJ. Monoclonal antibodies were covalently labeled with a HUH tag‐specific ssDNA sequence by click chemistry. An activated tyrosine within the HUH tag (‐O^–^) acts as nucleophile and attacks the phosphorous atom (yellow dot) in the ssDNA thereby generating a covalent phosphotyrosine linkage. b) Split intein‐mediated covalent coupling of single‐chain variable fragments (scFvs) to AAV2. NpuC was fused to the N‐terminus of receptor‐blinded VP2 and expressed in trans to the receptor‐blinded *rep*‐*vp1/3* plasmid for the production of NpuC‐displaying AAV2. NpuN was fused to the carboxy‐terminus of an scFv and separately produced in mammalian cell culture. The scFv was covalently coupled to Npu‐AAV2 by NpuC/NpuN‐mediated trans‐splicing thereby releasing the Npu intein.

So far, the modification of viral surface loops has mainly been exploited for the display of small peptides because larger protein domains were thought to impair the capsid structure and function. However, recently, VR IV has attracted interest for the incorporation of larger inserts. For example, nanobodies incorporated in VP1 of AAV2 retargeted viral particles to customized cell‐surface receptors.^[^ ^132^ ^]^ Similarly, this site has been exploited for the insertion of a T7 gene 2 protein (Gp2, 6 kDa), a nanobody (13 kDa), and a HUH tag (21 kDa) into the capsid of AAV‐DJ.^[^ ^133^ ^]^ HUH tags are derived from bacterial or viral endonucleases and form covalent phosphotyrosine linkages with ssDNA in a sequence‐specific, orthogonal manner.^[^ ^134^ ^]^ Displaying the *Escherichia coli*‐derived mobilization protein A (mMobA) HUH tag on AAV‐DJ enabled the covalent attachment of commercial antibodies that had been conjugated to ssDNA by copper‐free click chemistry (**Figure** **6**a).^[^ ^133^ ^]^

### 4.2 Modification of the N‐Terminus of VP2

An alternative strategy for displaying larger peptides and proteins on AAV is to fuse them to the N‐terminus of VP2, which leads to their exposure through the pore of the fivefold symmetry axis of the viral capsid (Figure 5). This approach was first used for the retargeting of AAV with a single‐chain variable fragment (scFv).^[^ ^135^ ^]^ Importantly, this study indicated the incompatibility of the reducing cellular environment with the incorporation of disulfide‐containing protein domains via the N‐terminus of VP2.^[^ ^136^ ^]^ AAV particles could only be produced as mosaics in the presence of all three unmodified VP proteins. Moreover, the infectious titer on target cells was low (1.9 × 10^2^ transducing units per mL).^[^ ^135^ ^]^ Nevertheless, the study pioneered the use of the N‐terminus of VP2 as an anchor point for protein domains intended for capsid display and retargeting. In addition, VP2 turned out to be dispensable for viral production and infectivity, and it could tolerate the addition of larger protein domains such as 30‐kDa GFP.^[^ ^137^ ^]^ The use of disulfide‐free affibody molecules^[^ ^138^ ^]^ and DARPins (designed ankyrin repeat proteins)^[^ ^139^, ^140^ ^]^ directed against cellular receptors opened the door to the targeting of distinct cell types in a highly specific and effective manner.^[^ ^15^, ^16^, ^17^, ^141^ ^]^ This approach was extended to disulfide‐containing scFvs by employing an intein‐based trans‐splicing approach (Figure 6b).^[^ ^142^ ^]^ To this end, the split *Nostoc punctiforme* (Npu) DnaE intein was exploited for a generic protein‐to‐AAV coupling approach. NpuC‐displaying AAV particles readily coupled to NpuN‐fused DARPins and scFvs, showed comparable targeting efficiency to the genetically fused counterparts, and exhibited reduced transduction of off‐target cells.^[^ ^142^ ^]^ Therefore, this method could be a universal approach for the coupling of targeting domains to the surface of AAV, thus broadening the spectrum of proteins that can be attached to the capsid.

In addition to the retargeting of AAV, the possibility of modifying the capsid surface has led to the emergence of new strategies for the development of vaccines. Previous concepts of delivering DNA vaccines via AAV^[^ ^18^, ^19^, ^20^, ^21^ ^]^ have been combined with VP2‐mediated antigen display on the viral capsid.^[^ ^143^ ^]^ The combined display and vector‐based expression of the *Mycobacterium tuberculosis* antigen AG85A resulted in a faster humoral immune response and did not require a booster dose for the induction of a memory response in mice.^[^ ^143^ ^]^

### 4.3 Serotype‐Hybrid AAV Capsids

Another approach for generating AAV variants with modified tropism is based on domain, peptide, and amino acid swapping between AAV serotypes (Figure 5).^[^ ^144^, ^145^, ^146^ ^]^ Chimeric virions generated in this way revealed capsid positions responsible for the differential tissue tropism and transduction kinetics of AAV serotypes. In addition to determining the structural properties crucial for transduction specificity and efficiency, these studies identified new positions that tolerate alterations and peptide insertions. For example, domain swapping and single amino acid exchanges between AAV2 and AAV8 revealed positions in the variable region VIII (VR VIII) of AAV8 that tolerate peptide insertions suitable for retargeting or the display of randomized peptide sequences.^[^ ^144^ ^]^ Moreover, receptor footprints on AAV serotypes were exploited for the directed development of chimeric AAV variants with altered tropism. Substituting a hexapeptide sequence located in the GH loop of AAV2 and containing residues R585 and R588 involved in heparan sulfate binding with those of other serotypes resulted in an AAV2/AAV8 chimera (AAV2i8) with a systemic transduction profile.^[^ ^147^ ^]^ Of note, AAV2i8 transduced cardiac and skeletal muscle with similar efficiency to AAV8, while showing markedly reduced targeting of the liver and a prolonged circulation half‐life in the blood.^[^ ^147^ ^]^ A similar approach was used to graft the galactose‐binding footprint from AAV9 onto AAV2 and AAV2i8.^[^ ^148^ ^]^ The resulting variants had unaltered tropism, but showed enhanced transduction and faster gene expression.^[^ ^148^ ^]^ In addition to receptor footprints, other capsid‐specific properties can also be exchanged between serotypes. AAV9, AAVrh.8, and AAVrh.10 are able to traverse the blood–brain barrier. This property was transferred to AAV1 by grafting the footprint from AAVrh.10 onto AAV1.^[^ ^146^ ^]^

### 4.4 Capsid Modifications Affecting Post Entry Steps

So far, the majority of rational capsid modifications have aimed at tailoring the specificity toward cellular receptors and/or protecting AAVs from NAbs. However, intracellular processes greatly affect the transduction efficiency of AAV‐based vectors. For example, the mutation of distinct serine, threonine, lysine, and tyrosine residues on the AAV capsid led to reduced ubiquitination and proteasomal degradation,^[^ ^149^, ^150^, ^151^ ^]^ accompanied with improved intracellular trafficking to the nucleus and increased transgene expression. It has also been shown that the neddylation and SUMOylation pathways are upregulated during intracellular AAV trafficking.^[^ ^152^ ^]^ Mutation of predicted target sites for these ubiquitin‐like modifiers led to AAV2 variants with enhanced transduction and transgene expression.^[^ ^152^ ^]^

## 5 Synthetic‐Biological Approaches to Control AAV Functions

### 5.1 Synthetic‐Biological Switches to Control Transduction

Synthetic biology uses molecular switches to control biological functions in response to specific internal or external stimuli.^[^ ^153^, ^154^ ^]^ The most prominent switches include protein–DNA, protein–protein, and protein–small molecule interactions that can be formed or dissociated in response to small molecules, enzymes, or light.^[^ ^155^ ^]^ This approach allows for the control of cellular processes such as transgene expression, receptor activation, or protein trafficking.^[^ ^156^ ^]^

#### 5.1.1 Chemically Controlled Transduction

**Figure 7:**
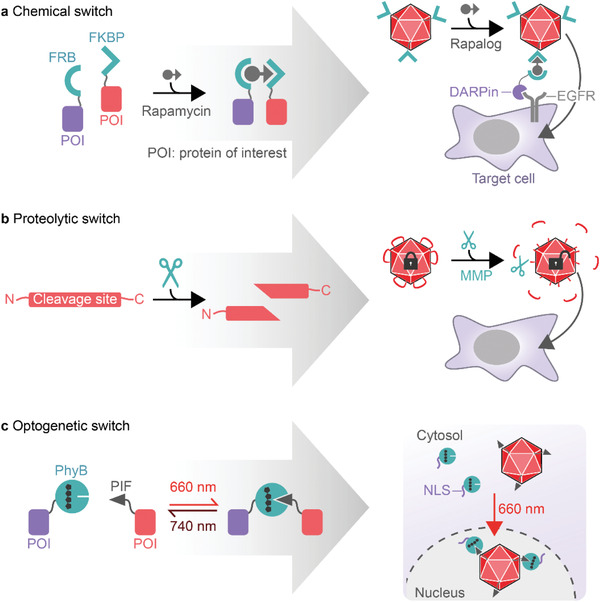
Synthetic‐biological switches for the control of AAV transduction. a) The interaction of proteins of interest (POI) can be controlled by chemically induced dimerization. In the presence of rapamycin, human FK‐binding protein (FKBP) binds to the FKBP‐rapamycin binding (FRB) domain of mTOR, thereby mediating the dimerization of attached POIs. This principle has been used to control the binding of FKBP‐displaying AAV2 to an EGFR‐specific DARPin fused to FRB. Rapalog‐controlled EGFR binding led to the transduction of EGFR‐expressing target cells. b) Sequence‐specific proteases can be used to release peptides or protein domains. The introduction of negatively charged peptide sequences into the HSPG‐binding loops of AAV blocked transduction of target cells. Insertion of MMP‐specific cleavage sites enabled the MMP‐triggered removal of the negatively charged peptides, which restored the infectivity of AAV. c) Optogenetic switches are used to control the interaction of POIs. Upon red‐light illumination (660 nm), phytochrome B (PhyB) fused to a nuclear localization signal (NLS) binds to PIF‐displaying AAV, supporting nuclear import, and thereby enhances transduction.

One example of a molecular switch is provided by the human FK‐binding protein (FKBP), which forms heterodimers with the FKBP‐rapamycin binding (FRB) domain of mTOR in the presence of rapamycin or analogs (rapalogs) thereof (**Figure** **7**a).^[^ ^157^, ^158^ ^]^ This chemically induced dimerization pair has been used for the pharmacologic control of gene expression,^[^ ^159^ ^]^ receptor activation,^[^ ^160^, ^161^, ^162^ ^]^ kinase activity,^[^ ^163^, ^164^ ^]^ protein trans‐splicing,^[^ ^165^, ^166^ ^]^ and epigenome editing.^[^ ^167^ ^]^ The toolbox of synthetic‐biological switches could be similarly applied for the design of AAV variants that can be controlled by external stimuli. For example, the fusion of FKBP to VP2 of receptor‐blinded (R585A, R588A) AAV2 enabled controlled interaction with an FRB‐tagged DARPin directed against the EGF receptor (Figure 7a).^[^ ^168^ ^]^ Addition of a rapalog triggered the interaction of AAV with the DARPin and targeted the vector to EGFR‐overexpressing cell lines.^[^ ^168^ ^]^

#### 5.1.2 Protease‐Triggered Transduction

In addition to chemical switches, enzymes can be used to control the interaction of protein domains by catalyzing the cleavage or formation of, e.g., peptide bonds. Among them, proteases are frequently used for synthetic‐biological applications and various sequence‐specific and orthogonal proteases have been well characterized. These proteases have been used to design cells that process input signals by means of synthetic proteolysis‐based circuits.^[^ ^169^, ^170^, ^171^ ^]^ Moreover, proteases have been exploited to control extracellular processes and to develop cell‐free sensor circuits and smart drug delivery vehicles.^[^ ^172^, ^173^, ^174^, ^175^, ^176^ ^]^ The latter use environmental cues to determine their site and/or time of action, such as the decreased pH or elevated levels of matrix metalloproteases (MMPs) that often characterize the microenvironment of tumor tissues.^[^ ^177^, ^178^ ^]^ pH‐ and MMP‐responsive delivery vehicles have the potential to specifically target such sites.^[^ ^179^ ^]^ A similar strategy has been developed for controlling AAV‐mediated gene transfer.^[^ ^129^ ^]^ The integration of negatively charged peptides into the heparan sulfate‐binding loop of AAV (between R585 and R588) effectively impaired the binding to heparin, which correlated with reduced cellular uptake. Insertion of MMP cleavage sites enabled the removal of the negatively charged peptide lock and the recovery of heparin binding and cell transduction (Figure 7b).^[^ ^129^ ^]^ While some MMPs are rather promiscuous, MMP‐7 and MMP‐9 show high cleavage site specificity. This enabled the design of mosaic viral particles responding to MMP‐7 and MMP‐9 with tunable AND gate Boolean logic.^[^ ^129^ ^]^ This strategy of integrating proteolytic biomarkers could endow AAV with the capability of locating and treating pathological sites such as tumor microenvironments. In a follow‐up report, the authors describe the impact of different tetra‐amino acid sequences on virion assembly, heparin binding, and transduction.^[^ ^180^ ^]^ Although both positively and negatively charged tetra‐amino acid motifs efficiently blocked heparin binding and transduction in the absence of MMP, only negatively charged inserts resulted in unimpaired production titers.^[^ ^180^ ^]^ This finding is consistent with the capsid fitness landscape described by Ogden et al.,^[^ ^33^ ^]^ who found that introduction of positively charged residues across all capsid positions interferes with viral assembly (see Section 3.7). Finally, MMP‐inducible AAV2 showed reduced inactivation by neutralizing antibodies and reduced off‐targeting to, e.g., the liver, while retaining its ability to target tumor tissue in an epithelial ovarian cancer mouse model.^[^ ^181^ ^]^

#### 5.1.3 Optogenetic Control of Transduction

Over the past decade, the steering of cellular functions with genetically encoded photoswitches has attracted considerable research interest. This field—termed optogenetics—exploits photoreceptors derived from, e.g., plants or bacteria as light switches to control intracellular and extracellular biological processes with unmatched temporal and spatial resolution.^[^ ^182^, ^183^ ^]^ Optogenetics enables fast, dose‐dependent, local, orthogonal, noninvasive, and reversible control of protein–protein interactions. Typically, illumination with a specific wavelength leads to a structural change of the chromophore and induces a conformational change in the photoreceptor, which exposes residues that are then accessible for the binding of interaction partners. Well‐characterized examples include cryptochrome‐2 (Cry2) and phytochrome B (PhyB), both derived from the wall cress *Arabidopsis thaliana*.^[^ ^184^, ^185^, ^186^, ^187^ ^]^ Cry2 homooligomerizes or forms heterodimers with CIB1 (cryptochrome‐interacting basic‐helix‐loop‐helix) upon blue‐light illumination and both interactions are reversed in darkness.^[^ ^184^, ^185^ ^]^ In contrast, PhyB is a red‐light‐responsive photoswitch that associates with its interaction partner PIF (phytochrome interacting factor) upon red‐light illumination (660 nm) and dissociates in response to far‐red light (740 nm).^[^ ^186^, ^187^ ^]^ The number of optogenetic switches is rapidly expanding, and spans the whole spectrum of visible light. A comprehensive overview of existing optogenetic tools including search engines for photoswitches and publications can be found at https://www.optobase.org/.^[^ ^182^ ^]^

Recently, the concept of optogenetics has been applied to control the transduction of AAV2.^[^ ^188^ ^]^ Gomez et al. exploited the nuclear transport of AAV, which is a rate‐limiting step during the transduction process, to develop red‐light‐tunable gene delivery.^[^ ^188^ ^]^ To this end, they displayed the phytochrome interaction partner PIF6 on AAV2 by fusing it to the N‐terminus of VP2. Expression of a nuclear localization signal (NLS) fused to PhyB enabled enhanced nuclear trafficking and thus gene delivery in response to red‐light illumination (Figure 7c). Since the use of light offers the possibility to adjust the dose, duration, and localization of induction, the system could be used to fine tune the expression strength and to spatially pattern gene expression in cultured cells.^[^ ^188^ ^]^ However, although red light shows good tissue penetration and thus is available for in vivo application, the requirement for modified cells expressing PhyB‐NLS limits the broad application of PIF6‐AAVs. This hurdle might be overcome by introducing an optogenetic switch operating extracellularly, e.g., by controlling the transduction at the level of receptor attachment.

### 5.2 Controlling AAV‐Delivered Gene Expression

**Figure 8:**
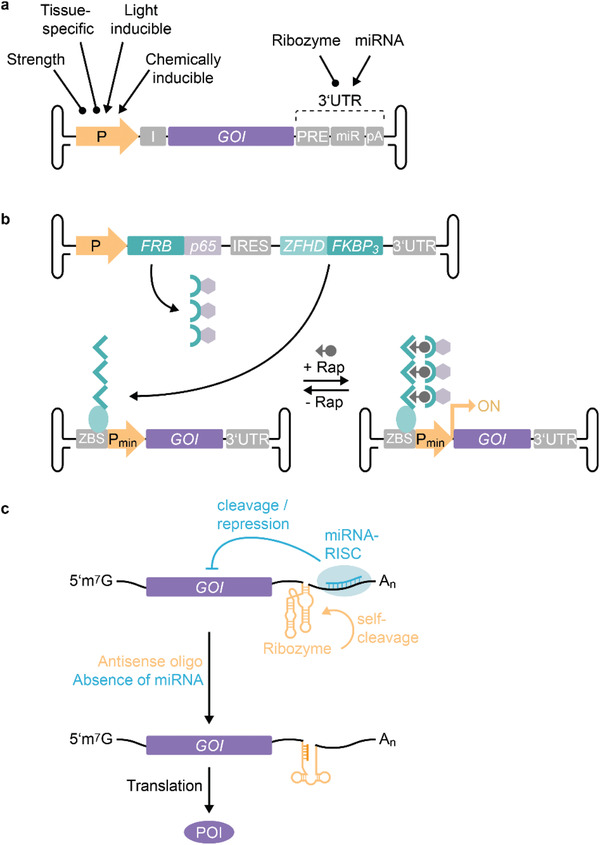
Strategies for controlling the expression of AAV‐delivered transgenes. a) The expression of vectored genes of interest (GOI) can be controlled at the transcriptional level by appropriately selecting the type of promoter. The choice of promoter strength and tissue‐specificity provides control of the transcription in target cells. In addition, synthetic promoters that can be controlled by chemicals or light enable the temporal and spatial fine‐tuning of gene expression. Posttranscriptional miRNA‐ or ribozyme‐based switches in the untranslated region (UTR) represent an additional possibility for controlling the level of gene products. b) Example of chemically induced AAV transgene expression. The system consists of two AAV vectors. The first produces a split transcription factor composed of a DNA‐binding zinc finger homeodomain (ZFHD) fused to three repeats of FKBP, and the p65 activator domain of NF*κ*B fused to FRB. The second vector expresses the GOI under the control of a synthetic promoter with upstream ZFHD‐binding repeats (zinc finger binding site, ZBS). In the presence of rapamycin (+ Rap), FRB binds to ZBS‐bound FKBP and thereby recruits the p65 activating domains to the promotor and stimulates the expression of the GOI. Withdrawal of rapamycin (‐ Rap) leads to the dissociation of FRB‐p65 and the downregulation of GOI expression. c) Translational control of AAV vector expression. Incorporation of miRNA targets into the UTR recruits the RNA‐induced silencing complex (RISC) to the mRNA and inhibits translation in cells expressing the miRNA. Differential miRNA expression profiles can be used to restrict the production of the protein of interest (POI) to target tissues. Alternatively, the incorporation of ribozymes into the UTR can be used to control the translation. Ribozyme self‐cleavage destabilizes the mRNA and effectively reduces the level of translation. Addition of antisense oligos inactivates the ribozyme and enables translation.

The expression of a transgene is controlled not only by the cellular uptake of the delivery vehicle and the trafficking of the genetic material to the nucleus, but also by coding and noncoding regulatory elements that act at the transcriptional or translational level (**Figure** **8**a). The ITR‐flanked expression cassette packaged into the AAV capsid can be modularly designed. Depending on the intended application, regulatory parts can be tailored to achieve transgene expression that is, e.g., broad, tissue‐specific, or time‐controlled. The most obvious regulatory element that can be chosen to spatially and/or temporally regulate the transcription is the promoter (Figure 8a). Strong, constitutive, and ubiquitously active promoters such as the cytomegalovirus (CMV) promoter or the hybrid CMV immediate early enhancer/*β*‐actin promoter (CAG)^[^ ^189^ ^]^ are often chosen in gene replacement therapies to achieve maximal transgene expression. This was the case for the approved drugs Glybera, Luxturna, and Zolgensma.^[^ ^8^, ^9^, ^39^ ^]^ Enhancer elements and post‐transcriptional regulatory elements (PRE) can further increase the efficiency of gene expression. For example, the commonly used woodchuck hepatitis virus PRE (WPRE)^[^ ^190^ ^]^ or intron sequences are thought to mediate interaction with the spliceosome, increasing the stability and/or nuclear export of the mRNA.

#### 5.2.1 Fine‐Tuning the Tissue Specificity of Transgene Expression

In cases where a specific tissue or cell type needs to be targeted, more sophisticated fine‐tuning of the gene expression could be advantageous and could be combined with the above‐described engineering of the viral capsid. Tissue‐specific promoters are usually weaker than constitutive promoters, but they add a layer of specificity to the gene expression profile and may help to reduce the effects of off‐target transduction. Additional strategies to increase the tissue specificity involve the exploitation of tissue‐specific expression profiles of miRNAs (Figure 8a,c). miRNAs act post‐transcriptionally by destabilizing the mRNA or inhibiting the translation and this can be used to restrict the expression of the transgene to specific tissues. For example, the target sequence for miRNA‐122 proved useful to suppress the expression of transgenes in the liver^[^ ^191^ ^]^ and was recently exploited to restrict CRISPR/Cas9‐mediated genome editing to the liver of mice.^[^ ^192^ ^]^ Combinational incorporation of different miRNA targets can further enhance cell and tissue specificity. For example, compared to healthy hepatocytes, miRNA‐122 and miRNA‐199a expression is downregulated in hepatocellular carcinoma (HCC), and the incorporation of the respective miRNA binding sites into AAV8 efficiently restricted the expression of a suicide gene to HCC cell lines.^[^ ^193^ ^]^ Other approaches use computational vector design to identify *cis*‐acting regulatory modules (CRMs) that enhance the gene expression in target tissues and thereby allow the use of lower vector doses, reducing the risk of T cell‐mediated immune responses against AAV. In a genome‐wide in silico analysis, Chuah et al. identified hepatocyte‐specific CRMs that contain signatures of evolutionarily conserved binding sites for transcription factors associated with high expression in the liver.^[^ ^194^ ^]^ Intriguingly, one CRM candidate increased the expression of the human clotting factor IX (hFIX) up to 100‐fold, depending on the strength of the promoter used. Treatment of nonhuman primates with a self‐complementary AAV9 coding for hFIX under the control of a potent liver‐specific promoter and the liver‐specific CRM led to high (20–35% of the normal levels) and sustained circulating levels of hFIX.^[^ ^194^ ^]^ A similar approach was used to enhance cardiac‐specific expression of genes delivered by AAV9.^[^ ^195^ ^]^

#### 5.2.2 Temporal and Adjustable Control of Transcription

In addition to regulating the strength and tissue specificity of transgene expression, the time of transcriptional induction or repression can also be adjusted. Over the past decade, synthetic biology has seen major developments in the field of controlled gene expression. Many chemically and optogenetically controlled tools have been developed and characterized, and enable excellent control and fine adjustment of gene expression.^[^ ^196^ ^]^ For example, bacterial repressor proteins, yeast transcription factors, and human hormone receptors have been exploited to develop eukaryotic gene switches that respond to clinically licensed antibiotics, flavonoids, amino acids, hormones, and other small molecules.^[^ ^155^ ^]^ The spectrum of transcriptional control elements has been further broadened by the introduction of chemically and optogenetically controlled protein–protein switches (see Section 5.1). The incorporation of synthetic, stimulus‐responsive transcription factors, and the corresponding promoters into the design of AAV vectors has resulted in tightly controlled and timely transgene expression in mice, rodents, and nonhuman primates (Figure 8b).^[^ ^197^, ^198^, ^199^ ^]^ Rivera et al. pioneered the use of humanized gene switches for controlling AAV‐delivered expression of human growth hormone and erythropoietin (Epo) in mice and primates, respectively.^[^ ^200^, ^201^ ^]^ Their system comprises three components of a rapamycin‐inducible gene expression system, packaged into two AAVs. One vector constitutively expresses a DNA‐binding zinc finger homeodomain (ZFHD) fused to three repeats of FKBP, and FRB fused to the activation domain of the p65 subunit of human NF*κ*B (Figure 8b). The second vector controls the expression of the therapeutic gene via a minimal interleukin‐2 (IL‐2) promoter with 12 upstream binding sites for ZFHD. Upon intramuscular co‐injection, rapamycin administration mediates the recruitment of FRB‐p65 to DNA‐bound FKBP and induces expression of the transgene (Figure 8b).^[^ ^200^, ^201^ ^]^ The system enables temporally controlled, rapamycin‐dose‐dependent, and reversible adjustment of protein expression. Notably, long‐term evaluation of Epo expression in rhesus macaques over 6 years suggested that a single injection was sufficient to achieve sustained regulated gene expression. Moreover, a combined AAV vector coding for all three components of the rapamycin‐inducible expression system proved to be functional as well.^[^ ^200^ ^]^ These studies underline the clinical feasibility of one‐time AAV administration to achieve long‐term control of therapeutic gene expression. The spectrum of synthetic‐biological gene switches for controlling the expression of AAV‐delivered genes was further extended by the introduction of deactivating systems. Fluri et al. used the macrolide‐responsive E.REX system^[^ ^202^ ^]^ in different configurations for erythromycin‐triggered shut‐off or induction of gene expression in mammalian cells and mice.^[^ ^203^ ^]^ The expression of both intracellular and secreted proteins could be reversibly adjusted in a time‐ and dose‐dependent manner.^[^ ^203^ ^]^ This concept was further combined with spatial, miRNA‐based regulation, or optogenetic control of a synthetic transcription factor.^[^ ^204^, ^205^ ^]^

#### 5.2.3 Posttranscriptional Control

Although the synthetic‐biological toolbox of transcriptional regulators is constantly growing, the packaging capacity of AAV (4.7 kb) limits the generic applicability in the design of vector genomes. Different strategies have been developed to enable the delivery of larger gene constructs, including overlapping dual vectors or trans‐splicing dual vectors.^[^ ^206^, ^207^, ^208^ ^]^ However, these methods usually require the co‐transduction of target cells and pose the risk of generating unrecombined or misrecombined structures.^[^ ^208^, ^209^ ^]^ A recent study employed a split intein approach for the expression of large transgenes, which resulted in more efficient protein reconstitution compared to dual AAV vectors.^[^ ^210^ ^]^ However, intein‐mediated trans‐splicing requires careful design of the split constructs and the inclusion of regulatory expression elements in all AAV vectors. Moreover, possible side effects derived from incomplete or non‐trans‐spliced proteins and excised inteins need to be evaluated. Another strategy for the packaging of larger transgenes is the use of shorter regulatory elements. In the context of inducible gene expression, an RNA‐based switch was recently proposed as an alternative to synthetic transcription factor/promoter elements.^[^ ^211^ ^]^ RNA‐based switches are considerably smaller than the expression cassette for a synthetic transcription factor and they do not code for potentially immunogenic proteins. Zhong et al. engineered a ribozyme that was inserted into the 3’UTR of a luciferase gene, where it efficiently destabilized the transcript.^[^ ^211^ ^]^ Addition of an antisense morpholino inactivated the ribozyme and rescued luciferase production (Figure 8c). The system was applied to control the expression of an AAV‐delivered transgene in mice and showed excellent morpholino‐responsiveness (200‐fold induction); it could be repeatedly activated, and was inducible over 20 months after AAV administration.^[^ ^211^ ^]^ The development of additional RNA‐based switches that show a comparable regulatory range could foster new approaches for alternative and multiplexed control of transgene expression, which could optionally be combined with the above‐described regulatory elements.

Strategies to spatially and/or temporally control the expression of a packaged transgene have great potential to be combined with engineered capsid variants. Developments in both capsid design and synthetic gene switches can be expected to advance gene delivery strategies.

### 5.3 Hybrid Viral Vectors

The search for optimal gene delivery vehicles has focused not only on the engineering of independent virus species, but also on combining advantageous features of different virus family members or even virus orders. In the case of AAV, different features such as the low genotoxicity of its recombinant vector genome, or the cellular uptake and endosomal escape mechanisms have been exploited for the development of hybrid vectors.

#### 5.3.1 Parvovirus Hybrids

The successful cross‐packaging of rAAV genomes into the parvorvirus B19 was reported more than 20 years ago.^[^ ^212^ ^]^ With the aim of developing parvoviral vectors for the treatment of hemoglobinopathies, Ponnazhagan et al. exploited B19's efficient tropism for human erythroid hematopoietic progenitor cells and packaged rAAV genomes into B19 capsids.^[^ ^212^ ^]^

**Figure 9:**
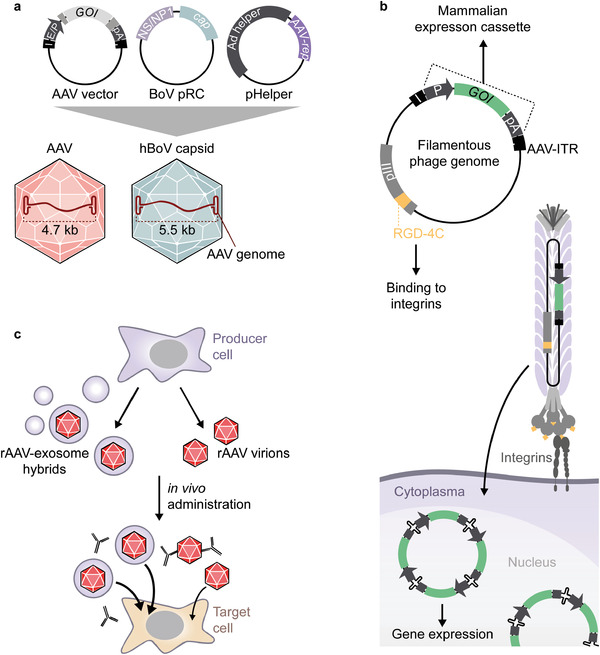
Hybrid AAV vectors for gene delivery. a) Cross‐packaging of an AAV genome into human bocavirus. Cross‐packaging is achieved by triple‐transfection of producer cells with AAV vector plasmid, a plasmid encoding the *rep* (NS/NP1) and *cap* genes of bocavirus (BoV pRC), and an adenoviral helper plasmid harboring the AAV *rep* gene (pHelper). The resulting hybrid viral particle has a packaging capacity of ≈5.5 kb.^[^ ^213^ ^]^ b) AAV‐phage hybrid (AAVP) for the transduction of mammalian cells. A mammalian expression cassette flanked by AAV‐ITRs is inserted into the intergenic region of the filamentous phage fd‐tet. An integrin‐binding RGD‐4C peptide is incorporated into the coat protein pIII. Upon uptake by integrin‐expressing cells, the AAV‐ITRs mediate concatemer formation and the stable expression of the transgene.^[^ ^215^ ^]^ c) AAV‐exosome hybrid. During AAV production, AAVs encapsulated by or associated with exosomes are released from producer cells. These hybrids are less prone to NAb inactivation and possess enhanced transduction efficiency compared to “naked” AAVs.^[^ ^229^, ^230^, ^231^ ^]^

Similarly, rAAV genomes have been incorporated into bocaparvoviruses. For example, Yan et al. cross‐packaged rAAV2 genomes into the capsids of the human Bocavirus‐1 (hBoV1), which resulted in efficient transduction of polarized human airway epithelial (pHAE) cells.^[^ ^213^ ^]^ Interestingly, bocaparvoviruses have an ssDNA packaging capacity of ≈5.5 kb, which permits the introduction of larger AAV expression cassettes (**Figure** **9**a). The Grimm lab extended the repertoire of cross‐packaged bocaparvoviral vectors to hBoV2‐4 and the Gorilla GBoV.^[^ ^214^ ^]^ In addition to pHAE cells, these hybrid vectors are also able to transduce other cell types, such as primary human hepatocytes, skeletal muscle cells, and CD4^+^ T cells. Moreover, capsid shuffling of hBoV1‐4 and GBoV generated a library with the potential to evolve next‐generation BoV variants featuring, e.g., desired tropism and efficient NAb evasion.^[^ ^214^ ^]^

#### 5.3.2 AAV‐Phage Hybrids

rAAV genomes can be packaged not only into capsids of related parvoviruses, but also into bacteriophages. Phages typically do not have any tropism for human cells but this capability can be introduced by the display of targeting ligands. To enable subsequent gene expression, eukaryotic regulatory elements are required. This was achieved by harnessing the compatibility of the ssDNA genomes of AAV and the M13‐derived phage dt‐tet.^[^ ^215^ ^]^ Insertion of rAAV expression cassettes into the intergenomic region of M13 phages displaying the RGD‐4C peptide enabled ligand‐targeted gene delivery to mammalian cells (Figure 9b). The AAV‐ITRs in the so‐called AAVP (AAV/phage hybrids) remained functional with respect to, e.g., the generation of recombinant AAV particles or concatemer formation in AAVP‐transduced cells.^[^ ^215^ ^]^ Upon systemic administration, the integrin‐targeted AAVP efficiently transduced different tumors in mouse xenograft models without off‐target delivery to, e.g., liver tissue. AAVP armed with the herpes simplex virus thymidine kinase (HSVtk) was used for both PET imaging with a radiolabeled nucleoside analog and tumor killing by the administration of the prodrug ganciclovir.^[^ ^215^ ^]^ Recently, this AAVP platform was further developed to display additional peptide sequences on the major phage coat protein pVIII.^[^ ^216^ ^]^ A short positively charged Ala‐Lys‐Ala‐Ser peptide, e.g., reduced nonspecific protein adsorption and neutralization by antiphage antibodies. The pVIII‐mediated display of an endosomal escape peptide derived from influenza hemagglutinin markedly enhanced transduction efficiency of RGD‐4C‐AAVP.^[^ ^216^ ^]^

Another approach combined the packaging capacity of T4 phage heads (≈170 kbp of dsDNA) with the ability of AAV to be taken up by mammalian cells and to mediate efficient endosomal escape.^[^ ^217^ ^]^ To this end, AAV particles were attached to T4 heads via biotin‐avidin bridges. These T4‐AAV hybrids indeed mediated the uptake of the genetic material packaged in the T4 moiety. In addition to the genes packaged in AAV and T4, the T4 head could co‐deliver attached proteins into mammalian cells.^[^ ^217^ ^]^ The ability to simultaneously package DNA and to display protein cargos was exploited for the proof‐of‐concept development of combined DNA and protein vaccines against influenza and *Yersina pestis*.^[^ ^217^ ^]^ Interestingly, delivery of the genetic material via the T4 head led to a TH1‐biased immune response, presumably due to the prokaryotic origin of the DNA exhibiting nonmethylated CpG islands and thus provoking the activation of toll‐like receptors. In contrast, AAV‐mediated DNA delivery evoked a more balanced TH1 and TH2 response.^[^ ^217^ ^]^ This work describes a creative and interesting approach to the generation of new vaccines. The potential of the system, such as loading capacity for different genes, the maximal size of gene constructs that can be packaged, and the applicability to other therapeutic applications, still needs to be investigated.

#### 5.3.3 AAV‐Exosome Hybrids

As mentioned above, pre‐existing humoral immunity is one of the greatest challenges for AAV‐mediated gene therapy and has been addressed from various angles. Although forward‐ and backward‐directed evolution (see Section 3) is highly promising for the selection of capsid variants with mutated antibody epitopes, it does not solve the challenge of repeated AAV administration in the clinics. Other strategies aim at the reduction of the patient's anti‐AAV NAb titers. For example, immunosuppression,^[^ ^218^, ^219^ ^]^ or antibody reduction by plasmapheresis^[^ ^219^, ^220^ ^]^ and hemapheresis^[^ ^220^ ^]^ have shown some degree of success. Further methods for lowering the functional NAb titers include the administration of receptor‐blinded, empty capsid decoys capable of capturing anti‐AAV NAbs^[^ ^221^ ^]^ and the less specific approach of administering IgG‐cleaving proteases.^[^ ^222^, ^223^ ^]^ For a detailed overview of immune responses to AAV and possible solutions, we refer the reader to the recent reviews by Nidetz et al.^[^ ^224^ ^]^ and Colella et al.^[^ ^225^ ^]^

An alternative strategy to protect AAV from NAbs is their encapsulation in a protective polymer shield^[^ ^226^, ^227^ ^]^ or in extracellular vesicles, i.e. exosomes, produced by eukaryotic cells (Figure 9c).^[^ ^228^ ^]^ The association of AAV with exosomes is a naturally occurring process. These AAV‐exosome hybrids, also known as vexosomes, are of great interest, because they show superior transduction efficiencies of different tissues and protection against NAbs.^[^ ^229^, ^230^, ^231^, ^232^ ^]^ Moreover, co‐expression of membrane‐tagged targeting ligands has been used to enhance the transduction efficiency in distinct tissues.^[^ ^228^, ^233^ ^]^ For example, fusion of the rabies virus glycoprotein (RVG) to the transmembrane domain of the platelet‐derived growth factor leads to its exposure on the surface of cells and exosomes, and was used for the construction of brain‐targeted vexosomes.^[^ ^233^ ^]^

In general, vexosomes show enhanced transduction and broad tropism for many tissues. These features might be advantageous for the treatment of monogenetic diseases affecting many cell types in the body. However, for therapies that require the specific targeting of distinct tissues or cell types, e.g., in the case of tumor targeting, additional strategies will be needed to minimize off‐target transduction.

## 6 Conclusions and Outlook

The development of reliable and safe tools for the delivery of genes is a pressing goal, e.g., for the treatment of monogenetic diseases and the development of new therapeutic approaches. In recent years, only a limited number of delivery vehicles qualifying for in vivo applications have been identified. Among them, AAV has attracted great interest because the safety and functionality of this vector are supported by the approvals of three AAV‐based therapies. However, despite these successes, we are still far from having a universally applicable and adaptable gene delivery vehicle. Major obstacles include the presence of neutralizing antibodies in the majority of people, the need to improve targeting of specific tissues and cell types, and the limited efficiency of gene transfer. The first issue plays a minor role in very young patients lacking a fully trained immune system and mostly being seronegative for anti‐AAV antibodies, or for the treatment of partially immune‐privileged sites such as the brain or the eye. Luxturna for retinal gene therapy and Zolgensma for the treatment of spinal muscular atrophy in children appear to be low‐hanging fruits in this respect. However, they may have opened the door to the development and approval of additional AAV‐based therapies. Therapeutic monoclonal antibodies were in a similar situation over 30 years ago. Orthoclone OKT3 was the first antibody approved for clinical use in humans,^[^ ^234^, ^235^ ^]^ although it was withdrawn from the market in 2010 due to the availability of alternative and safer therapies. Nevertheless, like Glybera, it pioneered a new therapeutic strategy. The murine origin of OKT3 could cause HAMA (human antimouse antibody) responses associated with mild to life‐threatening symptoms. Consequently, enormous efforts have been made to develop and manufacture humanized or fully human therapeutic antibodies.^[^ ^236^ ^]^ Today, these antibodies are successfully used for the treatment of different types of cancer, cardiovascular diseases, infectious diseases, or autoimmune diseases.^[^ ^237^ ^]^ Similarly, AAV‐based gene delivery offers huge potential for the development of new therapeutic approaches, such as gene replacement therapies, targeted virus‐directed enzyme prodrug therapy, or novel vaccination strategies. The approval of AAV‐based therapies has triggered an increased interest in the fundamental biology of this virus and in the engineering of new AAVs with improved functions. Both of these research topics go hand in hand and complement each other well. A deeper understanding of, e.g., cellular uptake mechanisms and intracellular trafficking will help to generate AAVs with enhanced transduction properties.

The future of AAV as therapeutic gene delivery vehicle strictly depends on the question of whether broad in vivo applicability of AAV can be achieved.

Additional factors that have to be taken into account, and which are often underestimated in academic settings, are the feasibility and expense of AAV production and downstream processing. Strategies that integrate all three major aspects—the engineering of AAV, the general in vivo applicability, and feasible production—are the most challenging yet potentially the most rewarding for achieving clinical breakthroughs.

Here, synthetic biology provides valuable tools and concepts to address major challenges. The incorporation of chemical and optogenetic switches into capsid designs opens the door to temporal and spatial precision targeting of tissues and cells. These designs could be further complemented by systems that restore the transduction capability in response to endogenous disease markers such as specific proteases or the low pH in the microenvironment of tumors. Such approaches might minimize off‐target transductions and thus side‐effects thereby increasing the efficiency of the viral vectors. Moreover, synthetic‐biological switches enable adjustable and time‐controlled gene expression, which could be a promising means to time, dose, and potentially minimize immune responses against the therapeutic gene. The challenge of preexisting immunity against AAV can be tackled by various approaches, which include the creation of novel or ancestral capsid variants devoid of current NAb epitopes, the engineering of stealth shieldings, or the development of completely new viral vector hybrids. A notable advantage of synthetic‐biological approaches is the modularity and flexibility of the design concepts, which facilitates the customization and tailoring of AAV variants.

The current clinical trials with bioengineered capsids are promising and will likely trigger a new era of AAV‐based research and development. An important factor that will impact on or even define future investigations is the integration of multiple disciplines for the design or evolution of next‐generation AAV vehicles. The value of interdisciplinary developments is clear in other scientific fields. The combination of molecular biology and cell biology techniques with (bio‐)chemical, mathematical, physical, or technical methods has resulted in the emergence of new disciplines in, e.g., synthetic biology,^[^ ^155^, ^156^, ^238^ ^]^ microsystems engineering,^[^ ^239^, ^240^, ^241^ ^]^ and materials science.^[^ ^179^, ^242^, ^243^ ^]^ A similar trend can be anticipated in the engineering of AAV variants to tackle current clinical challenges. Developments in synthetic biology could enable the engineering of AAV vehicles that go beyond the standard capsid modifications and expression cassette designs. Although it is questionable whether the current early designs will ever enter the clinic, they could be considered as a first step toward next‐generation gene‐delivery vehicles. The hope awakened by this small virus is big: synergies among basic research, rational and evolutionary designs, and synthetic‐biological engineering could foster the emergence of new approaches both to improve current therapies and to address thus‐far untreatable illnesses.

## Conflict of Interest

The authors declare no conflict of interest.
